# Long-term impact of oral surgery with or without amoxicillin on the oral microbiome-A prospective cohort study

**DOI:** 10.1038/s41598-019-55056-3

**Published:** 2019-12-10

**Authors:** R. K. Menon, A. Gomez, B. W. Brandt, Y. Y. Leung, D. Gopinath, R. M. Watt, W. Crielaard, K. E Nelson, M. G. Botelho

**Affiliations:** 1 International Medical University, Kuala Lumpur, Malaysia; 2 Faculty of Dentistry, University of Hong Kong, Hong Kong SAR, China; 3 J. Craig Venter Institute, La Jolla, San Diego, USA; 4 Department of Animal Science, University of Minnesota, Twin Cities, USA; 5 Department of Preventive Dentistry, Academic Centre for Dentistry Amsterdam, University of Amsterdam and Vrije Universiteit Amsterdam, Amsterdam, the Netherlands

**Keywords:** Antibiotic prophylaxis in dentistry, Oral microbiology

## Abstract

Routine postoperative antibiotic prophylaxis is not recommended for third molar extractions. However, amoxicillin still continues to be used customarily in several clinical practices worldwide to prevent infections. A prospective cohort study was conducted in cohorts who underwent third molar extractions with (group EA, n = 20) or without (group E, n = 20) amoxicillin (250 mg three times daily for 5 days). Further, a control group without amoxicillin and extractions (group C, n = 17) was included. Salivary samples were collected at baseline, 1-, 2-, 3-, 4-weeks and 3 months to assess the bacterial shift and antibiotic resistance gene changes employing 16S rRNA gene sequencing (Illumina-Miseq) and quantitative polymerase chain reaction. A further 6-month follow-up was performed for groups E and EA. Seven operational taxonomic units reported a significant change from baseline to 3 months for group EA (adjusted p < 0.05). No significant change in relative abundance of bacteria and β-lactamase resistance genes (TEM-1) was observed over 6 months for any group (adjusted p > 0.05). In conclusion, the salivary microbiome is resilient to an antibiotic challenge by a low-dose regimen of amoxicillin. Further studies evaluating the effect of routinely used higher dose regimens of amoxicillin on gram-negative bacteria and antibiotic resistance genes are warranted.

## Introduction

The oral cavity is the gateway to the digestive system and facilitates interaction between food, drugs, microbiota, salivary proteins and digestive enzymes. Transmission from health to disease is often associated with alterations in richness and diversity in the oral microbiome^1^. Some human diseases appear to be the result of perturbations in inherent microbial populations which result in an ecological imbalance, or *dysbiosis*^2^.

Antibiotic resistance is a major public health issue. Clinical antibiotic over-prescription appears to play a substantial role in propagating antibiotic resistance^3^. In the US alone, outpatient antibiotic prescription accounts for 30% of the estimated 50% of inappropriate antibiotic prescriptions^4^. Among those prescribed, amoxicillin is one of the most overly prescribed antibiotics in the US along with azithromycin^5^.

Amoxicillin is frequently prescribed in dentistry for third molar tooth surgery to prevent post-treatment infections^6–8^. However, two recent systematic reviews and meta-analyses has reported that amoxicillin when used alone is not effective in preventing post-surgical infection after third molar surgery^9,10^. Another systematic review assessing antibiotic use for tooth extractions, has reported on a range of clinical postoperative problems like local signs of infection, dry socket, pain, fever, swelling, as well as adverse effects of associated antibiotic use, like nausea and diarrhea^11^. Moreover, there are other problems associated with the use of antibiotics on the oral cavity which are not restricted to clinical outcomes, and include impacts on the oral microbiome^5,12^. Hence, there is a need to assess the long-term impact of amoxicillin use for third molar surgery on the oral microbiome and resistome.

The aim of the current study was to assess microbial changes and prolonged impact of amoxicillin use on the salivary microbiome in patients who received amoxicillin after surgical extractions compared to those who did not receive the antibiotic after extraction. Both patient groups were compared against healthy controls.

## Results and Discussion

A pilot study (P) was performed first to assess the changes in the bacterial profile and to identify resistance genes which demonstrate a significant shift in the oral microbiome of patients who undergo third molar surgery with or without antibiotic treatment. A shotgun metagenomic approach was chosen to comprehensively review all bacteria and resistance genes from the same salivary sample. After identifying the trends for change in the oral microbiome from the pilot study (P), a follow-up study (F) was performed to validate the findings of the pilot study on a larger sample size. 16S rRNA gene sequencing was employed here to study the bacterial shift and QPCR of TEM antibiotic resistance genes was performed, based on the findings from the pilot study.

The pilot study (P) had two groups (group XA-extraction with amoxicillin and group X-extraction without amoxicillin). The follow-up study had three groups; (group EA-extraction with amoxicillin, group E-extraction without amoxicillin and group C-control group without extraction or amoxicillin).

**Figure 1:**
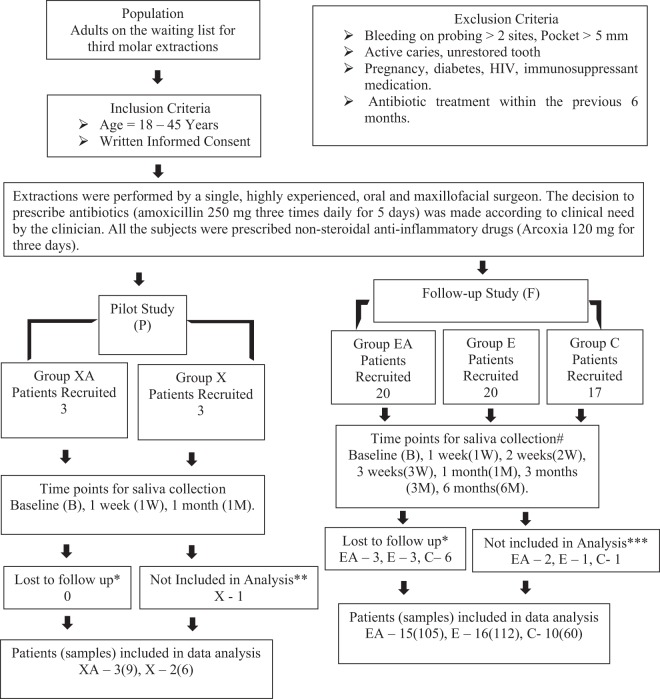
Study Design. Pilot Study (P), XA-Extraction with Antibiotics, X-Extraction without Antibiotics. Follow-up Study (F), EA-Extraction with Antibiotics, E-Extraction without Antibiotics. c. C-Control group. *Patient did not report for all appointments, **Patient had post-treatment infection and was prescribed augmentin on the 5^th^ day. ***Samples in any one-time point (out of 7) did not pass quality test to be included for sequencing in a single run. For Group C samples were collected only till 3 months.

The study flow is summarized in Fig. 1. The reporting of this study conforms to the STROBE statement. The demographic variables for the patients and the study variables are summarized in Table 1. Studies were planned without influencing treatment decisions to evaluate the impact of the standard practice of antibiotic use, or the avoidance of it, by clinical dental practitioners; therefore, no randomization was performed. We recruited only adults between the age group of 18–45 years for our study as this reflected the age group experiencing most complications after third molar extractions^13^. Exclusion criteria for periodontitis and caries were to minimize bias in the microbiome analysis, as subjects with these diseases have been shown to have distinct profiles for the salivary microbiome^14,15^.

**Table 1:** Demographic variables of the patients and study variables.

Variables	Pilot Study (Group X and XA)	Confirmatory Study (Group E and EA)	Confirmatory Study (Group C)
Age	19.75 (0.3)	22.3(1.2)	22.9(4.0)
Percentage of Females	40	64.5	70
Site(R:L)	40	50	NA
Number of teeth extracted	5	35	NA
Number of patients in whom two teeth were extracted in the same appointment	0	4	NA
Number of salivary samples analyzed (Time points)	15 (3)	217 (7)	60 (6)
Sequencing Technique	Shotgun Sequencing (Illumina HiSeq. 2500)	16S r RNA gene sequencing (Miseq), qPCR for TEM 1 genes.	16S r RNA gene sequencing (Miseq), qPCR for TEM −1 genes.

Pilot Study (P), a. XA-Extraction with Antibiotics, b. X-Extraction without Antibiotics. Follow-up Study (F), a. EA-Extraction with Antibiotics, b. E-Extraction without Antibiotics. c. C-Control group. NA – Not applicable since Group C did not undergo extraction or antibiotic treatment. qPCR – Quantitative polymerase chain reaction.

### Pilot study (P)

Bacterial shift and antibiotic resistance genes changes were assessed by shotgun metagenomic sequencing (Illumina-Hiseq) in the pilot study (P). 18 salivary samples from six patients over three time-points; baseline, one-week (1 W) and one-month (1 M), generated an average of 12008254 raw reads. After quality filtering and removal of human reads, an average of an average of 4518318 reads remained. The data was randomly subsampled to 922000 reads corresponding to the sample containing the minimum read count. Out of the six patients who were initially recruited for the pilot study, one patient who reported with post-surgical infection and was prescribed augmentin at the 5^th^ day, was excluded from further analyses regarding the impact of amoxicillin treatment.

**Figure 2:**
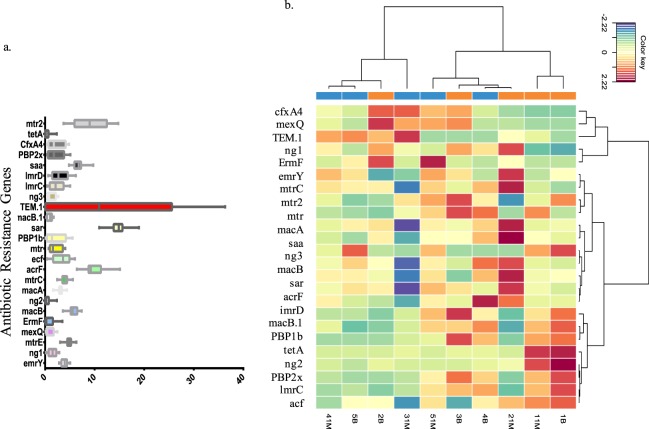
Pilot Study (P). Graphical representation of the relative abundance of antibiotic resistance genes in the five patients in pilot study (P) depicted for the baseline and the one month timepoint. TEM-1-beta-lactamase. Patient number. Timepoint (B-Baseline, 1M-One month). (**a**) Relative abundance of antibiotic resistance genes in the 15 samples collected from the five patients in the pilot study. (**b**) Heatmap of the antibiotic resistance genes in each sample collected in the pilot study. *Antibiotic resistance genes cfxA4-CfxA beta-lactamase, mexQ-subunit of efflux pump conferring antibiotic resistance (part of)MexPQ-OpmE, TEM-1-beta-lactamase, Ng-Neisseria gonorrheae mutant porin PIB (por) with reduced permeability to antibiotic, ermF-confers resistance to erythromycin, emrY- emrY is a multidrug transport that moves substrates across the inner membrane of the Gram-negative *E. coli*. mtr-mtrC is the membrane fusion protein of the MtrCDE multidrug efflux complex.macA- membrane fusion protein that forms an antibiotic efflux complex with MacB and TolC, saa- Staphylococcus aureus rpoB mutants conferring resistance to rifampicin, sar- Staphylococcus aureus gyrB conferring resistance to aminocoumarin, macB- ATP-binding cassette (ABC) transporter that exports macrolides with 14- or 15- membered lactones. It forms an antibiotic efflux complex with MacA and TolC, acrF- subunit of efflux pump conferring antibiotic resistance to ampicillin, ciprofloxacin and tetracycline, lmrC, lmrD- mrD is a chromosomally-encoded efflux pump that confers resistance to lincosamides in Streptomyces lincolnensis and Lactococcus lactis, PBP1b,2x -strpetococcus pneumoniae conferring resistance to amoxicillin, tetA- ABC transporter that confers resistance to tetracycline and tigercycline.

The Shannon Diversity Index (H) evaluates the species diversity by taking into consideration both the abundance and evenness of the OTUs present^16^. For each group, the shift in bacterial diversity from baseline to each of the subsequent time points (1 W and 1 M) was not statistically significant (p > 0.05, Anova, Tukey HSD). Compared to the baseline, at the 1 M time point, there was an increase in the relative abundance of *Proteobacteria* in Group XA but not in Group X. *Actinobacteria, Bacteriodetes and Firmicutes* showed a decrease in relative abundance from baseline to the 1 M time point for both groups. The relative abundance at the genus level are depicted as a table in Supplementary material, Table 1. None of the genus level changes from the baseline were statistically significant (Wilcoxon test, p > 0.05). However, the relative increase in *Proteobacteria* in the group treated with antibiotics prompted a further investigation in a larger cohort as explained in the follow-up study. Shotgun metagenomic techniques help in the evaluation of the whole genomic content in a sample and hence enabled us to evaluate the antibiotic resistance genes in the salivary samples. The class of antibiotic resistance genes which showed the largest magnitude of change in relative abundance among all the five patients between the baseline and the 1 M time point was TEM-1 (Fig. 2a). The changes from baseline to each time point for each patient (p > 0.05, Wilcoxon Signed Rank test) and when comparing Group XA to Group X were not statistically significant (p > 0.05, Mann-Whitney U test). Individualized changes in the resistance genes for all patients are visualized in (Fig. 2b). The presence of beta-lactamase resistance genes in high magnitude in patients treated with amoxicillin was an interesting observation in this small group of patients and hence was investigated further in the follow-up study.

Even though the shotgun metagenomic approach enabled us to study the bacterial profile and the resistance genes simultaneously the use of the same approach on a larger sample size was restricted by the associated cost. Hence, a follow-up study with a larger cohort to further investigate the sequential changes in the pilot study, particularly in relation to the bacterial shift and change in TEM-1 antibiotic resistance genes was planned employing 16S rRNA gene sequencing and QPCR respectively.

The complementary use of different techniques in the pilot study (P) and the follow-up study (F) is an efficient technique to investigate the microbial and antibiotic resistance changes in a larger sample size population. The more comprehensive shotgun metagenomic approach identifies the trends which can be further investigated in a follow-up study by a low-cost approach in a larger cohort for validation.

### Follow-up study (F)

16S rRNA gene sequencing (Illumina-Miseq) and quantitative PCR (qPCR) of TEM-1 genes were performed in the follow-up study (F). The temporal changes in the pilot study (P) for Group X which underwent extractions without antibiotics, prompted us to include a control group without extraction or antibiotic usage (Group C) in the follow-up study (F), to facilitate the comparison of sequential changes in the bacteria and resistance genes observed within groups EA and E.

After accounting for loss to follow up and discarding salivary samples with low quality, 277 samples from 41 patients over seven time-points; baseline, one week (1W), two weeks (2W), three weeks (3W), one month (1M), three months (3M) and six months (6M) were included in the final analysis. After the primer sequences were removed, tags without primers were 6159099 in total with 20530 tags per sample on average, and the average length was 421 bp.

**Figure 3:**
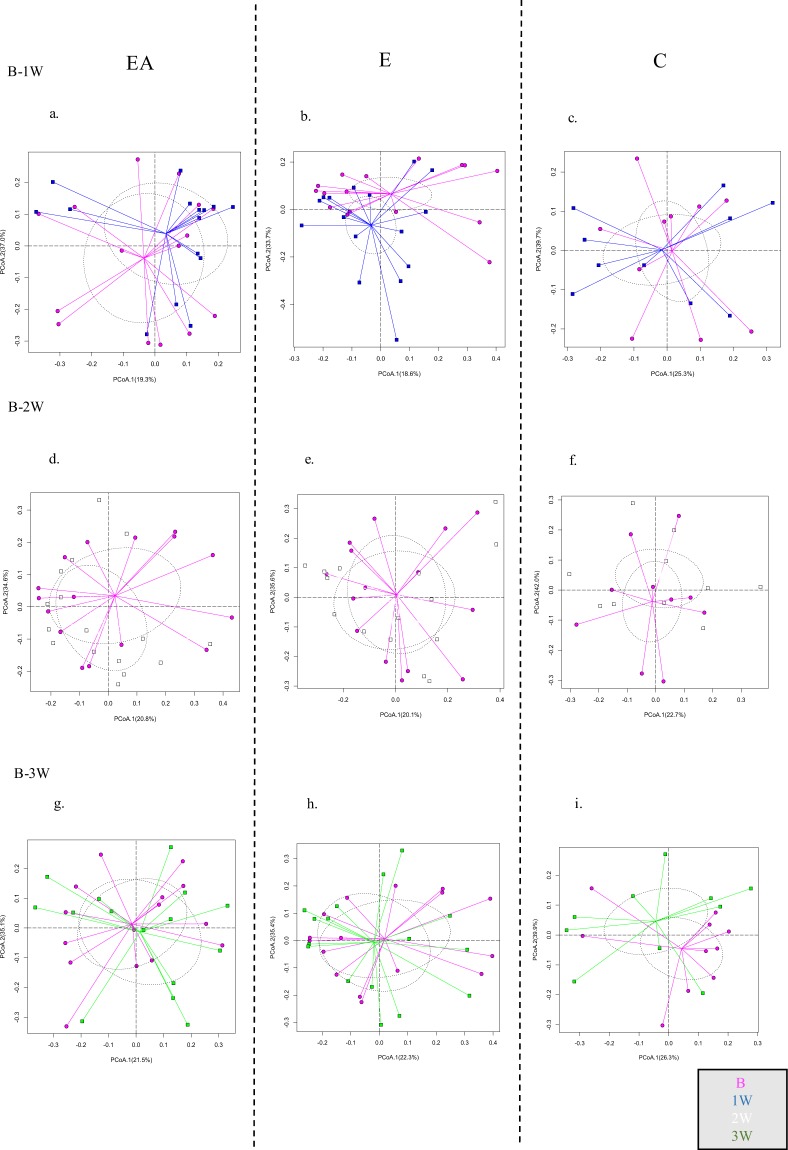
Follow-up Study (F). Principal coordinate analysis of microbial profiles comparing each timepoint to the baseline. (**a**) EA-Extraction with Antibiotics, (**b**). E-Extraction without Antibiotics. (**c**) C-Control group. B-Baseline, 1W-One week, 2W-Two weeks, 3W-Three weeks. (p > 0.05, ADONIS).

**Figure 4:**
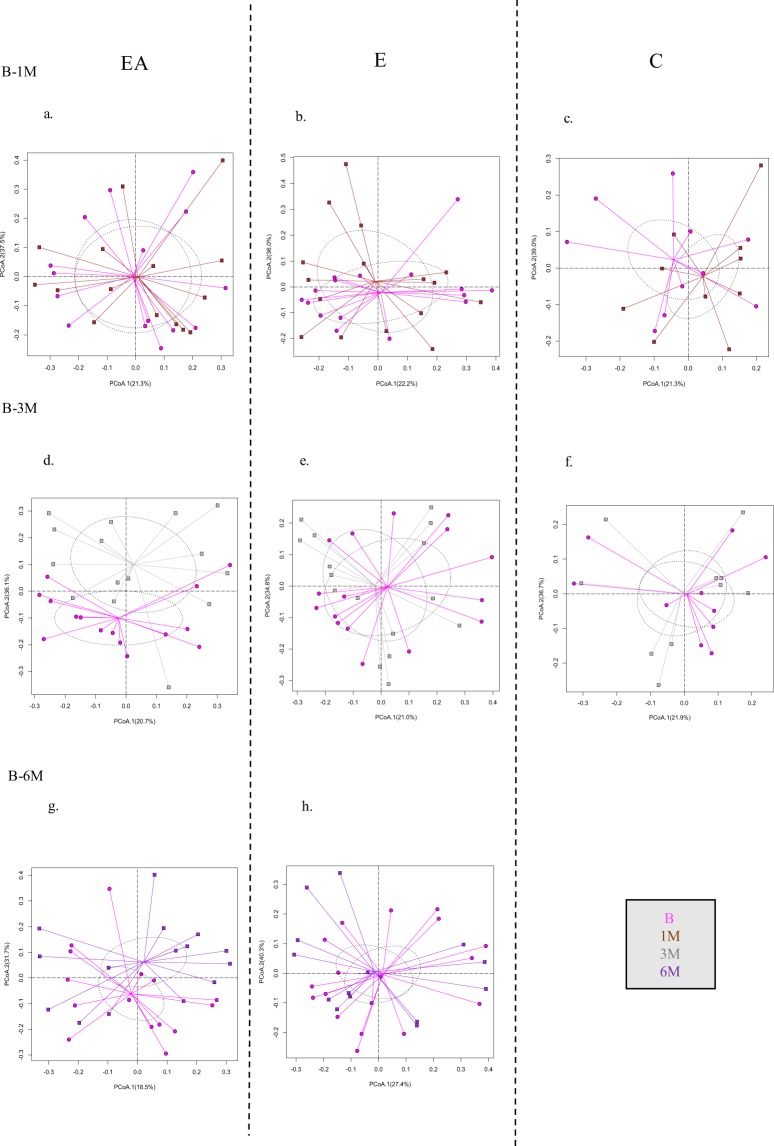
Follow-up Study (F). Principal coordinate analysis of microbial profiles comparing each timepoint to the baseline. (**a**) EA-Extraction with Antibiotics, (**b**). E-Extraction without Antibiotics. (**c**) C-Control group. B-Baseline, 1M-One month, 3M-Three months, 6M-Six months. (p > 0.05, ADONIS).

Principal Coordinates Analysis (PCA) was used to visually assess the dissimilarities in microbial community composition between samples for each group across all the time points (Figs. 3a–I and 4a–h.). None of the time point comparisons showed a statistically significant difference in microbial profiles for all the three groups (p > 0.05, ADONIS). We did not find a significant impact of antibiotic treatment on the overall microbial profiles for all groups across the seven time points as measured by the Bray-Curtis dissimilarity index.

**Figure 5:**
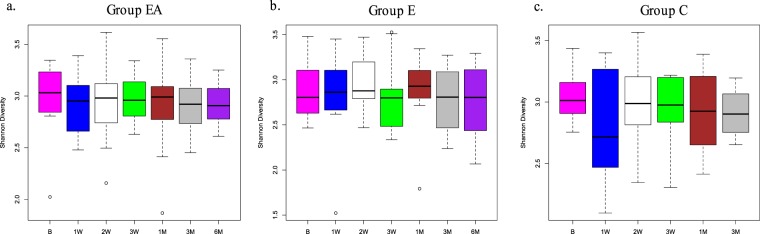
Follow-up Study (F). Shannon Diversity Index across all time points for all groups. (**a**) EA-Extraction with Antibiotics, (**b**). E-Extraction without Antibiotics. (**c**) C-Control group. B-Baseline, 1M-One month, 3M-Three months, 6M-Six months. (p > 0.05).

For Group EA, the Shannon Diversity Index (H) values were at a lower level after treatment, and the decline persisted up to 6 months (Fig. 5a). In contrast, Group E showed a more stable microbiome from baseline up to 6 months, with H values remaining at pretreatment levels or above throughout the time period (Fig. 5b). Within Group C, the H values observed throughout the study remained comparable to those determined at the baseline; with the exception of the 1 Week and 3 Month time points, which exhibited slightly lower mean diversity levels (Fig. 5c). However, the changes across the time points were not statistically significant for any of the three groups (p > 0.05, Anova, Tukey HSD). The sustained reduction of microbial diversity in saliva after amoxicillin use in healthy volunteers up to a period of 6 months has been recently reported in a study by Abeles *et al*.^5^. However, our study is the first to report this in the context of amoxicillin use for a dental procedure. Nevertheless, these results should be considered with caution, as higher fluctuations in diversity and lower values for mean diversity were observed for the control group (with no extractions or antibiotic treatment) at the 1 Week and 3 Month time points. This result is strikingly similar to the previously mentioned study where healthy volunteers who received a placebo instead of amoxicillin showed a similar lowering of diversity levels^5^. The absence of any significant change in the microbial profiles as well as diversity for all groups is in accordance with previous evidence that the salivary microbiome is more resilient to changes to systemic use of amoxicillin when compared to the gut microbiome^5,12^. It does appear that overall temporal community changes after antibiotic treatment with respect to changes in diversity and dissimilarity indices are more evident for body sites other than the oral cavity^17^.

The relative abundance of bacterial phyla across six time points for all the three groups was calculated. Most notably, Group EA showed a 60% decrease in the relative abundance of *Actinobacteria* from baseline to 1 Week, which then persisted as a 50% decrease at the six-month time point. *Bacteriodetes* and *Fusobacteria* exhibited a 20% decrease from baseline at 2 Weeks. The decrease in *Fusobacteria* continued further to 40% at 6 months. However, levels of *Bacteriodetes* recovered to 5% below baseline at 6 months. There was a persistent increase in the relative abundances of *Proteobacteria* and *Firmicutes* from baseline up to 6 months (Supplementary material, Fig. 1a). A 30% increase in the levels of *Proteobacteria* at one week continued as a 25% increase from baseline at 6 months. *Firmicutes* showed a 20% increase from baseline to 2 weeks after antibiotic treatment which increased to 30% at 6 months. However, Group E and Group C did not show consistent trends in the longitudinal changes of the bacterial phyla over time; with all phyla either remaining at or above the pretreatment levels throughout the study, or attaining the baseline levels by 3 months (Supplementary material, Fig. 1b,c). Our study reports persistently elevated levels of *Proteobacteria* up to the 6 Month time point for the group treated with amoxicillin; which is in clear contrast with both the other groups where the levels of *Proteobacteria* remained close to the baseline levels throughout the observation period. Lazarevic *et al*. have previously reported a similar increase in the salivary levels of *Proteobacteria* after amoxicillin treatment. The aforementioned study also showed a decrease in phylum *Actinobacteria*, which almost reverted back to baseline levels by the third week after treatment^18^. However, our study shows a further reduction in *Actinobacteria* at the 1 Month time point which persisted up to 6 months. These findings indicate a longer and sustained impact of amoxicillin treatment in saliva.

To further test the community changes across time points, we used the “wilcox.test” function in R to compare each time point to baseline. Out of 303 OTUs overall, 67 OTUs (23%) showed a significant change in their relative abundance from baseline over a period of three months for Group EA compared to 29 OTUs (10%) for Group E (unadjusted p < 0.05, Wilcoxon Test). For Group C only 13 OTUs (5%) showed a significant change from baseline over 3 months (unadjusted p < 0.05, Wilcoxon Test). Including the additional time point, Group EA showed a significant change for 73 (24%) OTUs over 6 months compared to 32 (11%) for Group E (unadjusted p < 0.05, Wilcoxon Test) (Supplementary material, Figs. 3 and 4). After correction of false discovery rates using the function “p. adjust” in R, 7 OTUs showed a significant change from baseline for Group EA (Table 2) (Supplementary material, Fig. 4) (p < 0.05, Wilcoxon Test, fdr adjusted), whereas no OTUs showed a significant change from baseline to any time point for Group E and Group C (p > 0.05, Wilcoxon Test, fdr adjusted). After adjustment for false discovery rates, the number of OTUs showing a significant change are reduced considerably. However, the remaining significant changes were seen only in Group EA indicating that amoxicillin treatment after surgery has a differing impact to the other two groups.

**Table 2:** Number of OTUs showing a significant change from baseline for the group treated with antibiotics after extraction (EA) after adjustment for false discovery rates.

OTU (p < 0.05, Wilcoxon test, fdr adjusted)
Streptococcus_sp_oral_taxon_058
Dialister_invisus_oral_taxon_118
Cardiobacterium_hominis_oral_taxon_633
Capnocytophaga_sputigena_oral_taxon_775
Streptococcus_salivarius_oral_taxon_755
TM7_G.3_sp_oral_taxon_351
Campylobacter_rectus_oral_taxon_748

(p < 0.05, Wilcoxon Test, fdr adjusted).

In Group EA, *Aggregatibacter paraphrophilus* was detected at the 1 Month time point and persisted up to the 3 Month and 6 Month time points. We could not find any such OTU with persistence up to 6 months in Group E, or up to three months in group C. The appearance and persistence of (detectable levels of) *A. paraphrophilus* in Group EA is noteworthy. *A. paraphrophilus* is a bacterium found in the nasopharynx and the oropharynx which has been previously implicated in infective endocarditis^19^ as well as liver and brain abscesses (Ariyaratnam *et al*. 2010). The appearance and persistence of this bacterium only in the group treated with amoxicillin should be investigated further, particularly in relation to bacteremia after a future extraction in the same individuals.

At the 1 Month time point, none of the three groups showed an increase in fold changes of TEM-1 gene compared to the baseline. However, at the 3 Month time point, group EA showed a 1.08-fold increase in contrast to group E and group C, which exhibited no increases. At the 6 Month time point, group EA showed a 7.32-fold increase in TEM-1 gene from the baseline, compared to a 2.41-fold increase for Group E (Supplementary material, Table 2). The changes from baseline to each time point for each group were not significant (p > 0.05, Wilcoxon Signed Rank test). Similarly, no significant differences were observed when comparing respective TEM-1 levels between Group EA, Group E and Group C (p > 0.05, Kruskal Wallis test). In short, the subjects exhibited a high degree of inter-individual variation in their respective TEM-1 gene levels across the study period. Within the group treated with amoxicillin, we observed an elevation in Class A β-lactamase genes (TEM-1) at the 3 Month and 6 Month points, which was accompanied by a corresponding increase in the levels of (gram-negative) *Proteobacteria*. This may imply a change in the antibiotic resistance potential of the ‘oral resistome’ and therefore make the oral microbiota resistant to future antibiotic use^20^. The increase of such bacterial taxa has been observed to cause elevation in gut bacterial genes conferring resistance to penicillin, without antibiotic treatment^21^. Furthermore, oral bacteria have previously been shown to affect systemic health and have the potential to enter the bloodstream, to cause infections in sites other than the oral cavity^22–24^. The comparative elevation and persistence of TEM-1 gene in some individuals are clinically significant. TEM-1 genes are typically found in gram-negative bacteria and are responsible for ampicillin and penicillin resistance in *Haemophilus influenzae* and *Neisseria gonorrheae*. Extended spectrum β-lactamase activities that confer multi-drug resistance can be due to derivatives of TEM-1, TEM-2 or SHV-1, via mutations that alter the amino acid configurations^25,26^.

Only one patient reported with a post–operative abscess which subsided immediately by incision and drainage followed by antibiotic treatment with amoxicillin-clavulanic acid (Augmentin®). The long-term deleterious impact of antibiotic treatment is a factor that has to be weighed against the low incidence of postoperative infection after third molar surgery.

We acknowledge the absence of detailed information and analysis of potential confounding factors from lifestyle habits including diet, smoke, alcohol consumption in our study. However, the impact of these factors on the analysis might be limited as the analysis of the microbiome compares variations from the baseline for each group. Our study shows that the salivary microbiome is resilient to an antibiotic challenge by a low-dose (250 mg) regimen of amoxicillin. However, the differing and prolonged impact of amoxicillin use on the gram negative bacteria and β-lactamase resistance should be investigated further particularly with higher dose regimens of amoxicillin. When correlated with current evidence on the inefficacy of amoxicillin as a prophylactic antibiotic for third molar surgery^9,10^, the benefit of using amoxicillin to prevent postoperative infections after third molar surgery should be reassessed.

## Methods

### Subject recruitment and grouping

Patients on the waiting list for third molar extractions at the Prince Philip Dental Hospital, The University of Hong Kong, were recruited after obtaining written informed consent. Subjects with a history of antibiotic use in the previous 6 months or active caries or periodontitis were excluded from the study. Subjects were advised to refrain from eating and performing any oral hygiene procedures for at least two hours before sample collection. All subjects were provided with a standard toothbrush and toothpaste that did not contain antibacterial agents to be used throughout the course of the study, they were advised to refrain from using any mouth rinse. Patient compliance with antibiotic treatment was confirmed by checking the antibiotic tablet strips on recall appointments.

A pilot study (P) followed by a follow-up study (F) was performed. The demographic variables for the patients and the study variables are summarized in Table 1. The study flow is summarized in Fig. 1.

### Saliva collection, DNA extraction, sequencing and data analysis

Three milliliters of unstimulated saliva were collected and stored at −80 °C until DNA extraction was performed. The sample collection was performed in the Prince Philip Dental Hospital, Hong Kong and immediately stored in the freezer available on-site. DNA extraction was performed using the Qiagen DNA mini kit (QIAGEN, Hilden, Germany) and the extracted DNA was stored at −20 °C. Concentration testing was performed using a Micro Plate Reader (Qubit Fluorometer, Invitrogen) and sample integrity testing was performed by Agarose Gel Electrophoresis (1% agarose, 150 V, electrophoresis time: 40 min). All the samples with a concentration of less than 5 ng/microliter were excluded. Amplification of bacterial DNA was performed using PCR primers targeting the 16S rRNA gene V4 (319F-806R) and the products were purified with AmpureXPbeads (AGENCOURT). After quantification by real-time quantitative PCR (RT-qPCR) (EvaGreenTM)the qualified libraries were sequenced on the Illumina Miseq System using the PE300 reagent Kit.

#### Pilot study (P)

Libraries were constructed, validated and paired-end sequenced on a HiSeq. 2500 System (HiSeq SBS Kit V4, Illumina) for 150 bp reads. Sequence filtering and quality control of the shotgun metagenomic data were performed using Knead Data v.0.4.6.1 (https://bitbucket.org/biobakery/kneaddata). The reads passing Knead Data were randomly subsampled to 922,000 reads based on the sample with the lowest number of reads and were analyzed with MetaPhlAn2 for assessing taxonomic distribution^27^. Gene assembly was performed with metaSPAdes^28^, followed by gene prediction with Prodigal^29^. From the predicted genes the resistance gene annotation was performed utilizing the CARD database^30^.

#### Follow-up study (F)

Amplification of the bacterial DNA was performed using PCR primers 319 F and 806 R targeting the 16S rRNA gene V3 - V4 regions. Raw data was pre-processed to obtain ‘clean’ data according to a previously established procedure^31^. Clustering of the tags with a threshold of 97% was performed using UPARSE and unique representative sequences of each operational taxonomic unit (OTU) were obtained, followed by filtering out the chimera using UCHIME (v4.2.40). All tags were mapped to representative sequences using USEARCH GLOBAL and the tag numbers in each sample were summarized to the OTU abundance table^32^. Taxonomical classification of the sequences was performed using the Ribosomal Database Project (RDP)^33^ Classifierv2.2 trained on the Greengenes database^34^ using a confidence value of 0.8 as cutoff.

The TEM-1 antibiotic resistance gene was amplified using the primers bl2btemf (GCCTGCAGCAATGGCAACAA)/bl2btemr (TGGTCCTGCAACTTTATCCG). The 16S rRNA gene was amplified in each sample utilizing the primers described in a previous study^35^. All primers were synthesized using Sigma-Aldrich (USA). The cycling program was performed on an ABI Prism 7900HT (Applied Biosystems, USA) under the following conditions: 10 min at 95 °C, followed by 40 cycles of 15 s at 94 °C and 1 min at 60 °C. Further characteristics of the primers used in the study are described in Supplementary material, Figs. 1 and 2. The PCR mixture without template DNA was included in each run as a negative control. The TEM-1 gene abundance was normalized to the 16S gene count in each sample.

### Statistical analysis

Statistical analysis was performed using R statistical software (v 3.1.1) predominantly using the Vegan package (v 2.4–4) for multivariate statistics. Analysis of variance with Tukey post hoc test was used for multiple comparisons of bacterial diversity between multiple time points. Wilcoxon (paired) signed rank test embedded within the R package was used to test significant changes in each bacterial OTU and TEM-1 gene from baseline to each separate time point. Differences in the microbial profiles at the community level were tested with the ADONIS function (Vegan) based on Bray – Curtis dissimilarity indices. Mann-Whitney U test and Kruskal-Wallis test was used for intergroup comparisons in study (P) and study (F) respectively.

### Ethics approval and consent to participate

This study was approved by the Institutional Review Board of the University of Hong Kong/Hospital Authority Hong Kong West Cluster. IRB Reference Numbers: UW 15–334 (P), UW 15–482 (F). Written informed consents for participation were obtained from all participants in the study. All research was performed in accordance with relevant guidelines/regulations.

## Supplementary information

Supplementary material

## Data Availability

Illumina MiSeq amplicon sequences of 16S rRNA genes of bacteria are available in the NCBI Sequence Read Archive (www.ncbi.nlm.nih.gov/sra) as BioProject PRJNA586897. All other data is available in the Supplementary Material.
