# Supplementary material for: Long-term impact of oral surgery with or without amoxicillin on the oral microbiome-A prospective cohort study
Source: Sci Rep. 2019 Dec 10;9:18761. doi: 10.1038/s41598-019-55056-3 (PMC6904678; doi:10.1038/s41598-019-55056-3)
Supplement: Supplementary file 1 — Supplementary material [file 41598_2019_55056_MOESM1_ESM.docx]

**Supplementary Material**

**Long-term impact of oral surgery with or without amoxicillin on the oral microbiome-A prospective cohort study**

R.K. Menon^1,2^, A. Gomez^4^, B.W. Brandt^5^, Y.Y. Leung^2^, D. Gopinath^2^, R.M. Watt^2^, W. Crielaard^5^, K.E Nelson^3^, and *M.G. Botelho^2^

^1^ International Medical University, Kuala Lumpur, Malaysia.

^2^ Faculty of Dentistry, University of Hong Kong, Hong Kong SAR, China.

^3^ J. Craig Venter Institute, La Jolla, San Diego, USA.

^4^ Department of Animal Science, University of Minnesota

^5^ Department of Preventive Dentistry, Academic Centre for Dentistry Amsterdam, University of Amsterdam and Vrije Universiteit Amsterdam, Amsterdam, the Netherlands.

*Corresponding Author

**Fig 1.** Follow-up Study (F). Relative abundance of six dominant bacterial phyla in saliva. a. EA-Extraction with Antibiotics, b. E-Extraction without Antibiotics. c. C-Control group. B-Baseline, 1W-One week, 2W-Two weeks, 3W-Three weeks, 1M-One month, 3M-Three months, 6M-Six months.

Fig 2. Relative abundance of OTUs showing a significant change (FDR unadjusted) from baseline for the control group and the group which underwent extraction without antibiotics. E-Extraction without Antibiotics. C-Control group. B-Baseline, 1W-One week, 2W-Two weeks, 3W-Three weeks, 1M-One month, 3M-Three months, 6M-Six months.

Fig 3. Relative abundance of OTUs showing a significant change **(FDR unadjusted)** from baseline for the group EA-Extraction with Antibiotics. B-Baseline, 1W-One week, 2W-Two weeks, 3W-Three weeks, 1M-One month, 3M-Three months, 6M-Six months.


**Fig 4.** Relative abundance of OTUs showing a significant change from baseline for the group treated with antibiotics after extraction (EA) after adjustment for false discovery rates. (p > 0.05, Wilcoxon Test, fdr adjusted)

Table 1. Relative abundances at the genus level in the salivary microbiome as assessed by shotgun metagenomic sequencing in the pilot study (P).

| **Genus** | **1B** | **11W** | **11M** | **2B** | **21W** | **21M** | **3B** | **31W** | **31M** | **B** | **41W** | **41M** | **5B** | **51W** | **51M** |
| --- | --- | --- | --- | --- | --- | --- | --- | --- | --- | --- | --- | --- | --- | --- | --- |
| Actinomyces | 0.00 | 0.00 | 0.14 | 1.44 | 1.42 | 0.30 | 1.01 | 1.39 | 1.84 | 0.60 | 1.12 | 2.00 | 4.47 | 1.48 | 2.90 |
| Corynebacterium | 0.32 | 0.27 | 1.01 | 0.00 | 0.00 | 0.00 | 1.99 | 0.25 | 3.03 | 2.90 | 2.26 | 3.26 | 0.75 | 0.42 | 0.26 |
| Rothia | 13.39 | 11.65 | 9.31 | 24.09 | 14.30 | 23.31 | 14.67 | 8.15 | 11.90 | 18.29 | 15.12 | 14.09 | 7.88 | 5.20 | 8.41 |
| Nakamurella | 0.00 | 0.00 | 0.00 | 0.00 | 0.00 | 0.00 | 0.00 | 0.00 | 0.00 | 0.00 | 0.00 | 0.00 | 0.00 | 0.00 | 0.00 |
| Propionibacterium | 0.00 | 0.00 | 0.00 | 0.00 | 0.00 | 0.00 | 0.00 | 0.00 | 0.06 | 0.13 | 0.00 | 0.28 | 0.00 | 0.00 | 0.00 |
| Atopobium | 0.00 | 0.00 | 0.00 | 0.86 | 0.28 | 0.18 | 0.32 | 0.00 | 1.35 | 0.00 | 0.42 | 0.44 | 0.29 | 0.00 | 0.44 |
| Slackia | 0.00 | 0.00 | 0.00 | 0.00 | 0.00 | 0.00 | 0.00 | 0.00 | 0.00 | 0.00 | 0.00 | 0.00 | 0.00 | 0.24 | 0.00 |
| Porphyromonas | 13.32 | 12.30 | 19.00 | 0.04 | 2.69 | 4.22 | 5.86 | 12.94 | 4.73 | 3.53 | 1.07 | 6.16 | 0.00 | 11.92 | 7.19 |
| Tannerella | 0.00 | 0.00 | 0.00 | 0.00 | 0.00 | 0.00 | 0.00 | 0.00 | 0.00 | 0.22 | 0.25 | 0.35 | 0.00 | 0.00 | 0.00 |
| Alloprevotella | 0.08 | 0.09 | 0.47 | 0.26 | 2.23 | 0.26 | 0.23 | 0.41 | 2.56 | 0.39 | 2.65 | 0.27 | 3.06 | 0.69 | 1.38 |
| Prevotella | 1.51 | 1.11 | 1.17 | 24.19 | 21.92 | 6.33 | 21.15 | 7.01 | 17.44 | 3.51 | 14.32 | 12.35 | 10.88 | 9.94 | 21.72 |
| Capnocytophaga | 1.48 | 1.52 | 2.56 | 0.93 | 3.31 | 1.17 | 0.69 | 1.26 | 0.54 | 2.71 | 1.26 | 3.08 | 2.39 | 1.33 | 0.95 |
| Abiotrophia | 0.04 | 0.06 | 0.50 | 0.00 | 0.00 | 0.00 | 0.11 | 0.00 | 0.00 | 0.00 | 0.02 | 0.20 | 0.00 | 0.00 | 0.06 |
| Granulicatella | 2.43 | 2.01 | 3.10 | 1.12 | 0.36 | 1.98 | 1.32 | 1.08 | 1.27 | 1.19 | 0.59 | 0.73 | 1.35 | 1.42 | 2.45 |
| Lactobacillus | 0.00 | 0.00 | 0.00 | 0.00 | 0.00 | 0.41 | 0.00 | 0.00 | 0.00 | 0.00 | 0.00 | 0.00 | 0.36 | 0.00 | 0.00 |
| Lactococcus | 0.00 | 0.00 | 0.00 | 0.00 | 0.00 | 0.00 | 0.00 | 0.00 | 0.00 | 0.00 | 0.00 | 0.00 | 6.91 | 0.00 | 0.00 |
| Streptococcus | 20.24 | 11.50 | 5.79 | 8.75 | 3.95 | 11.51 | 8.40 | 7.90 | 11.54 | 10.99 | 11.75 | 10.15 | 9.77 | 8.08 | 10.73 |
| Eubacterium | 0.00 | 0.00 | 0.00 | 0.00 | 0.00 | 0.00 | 0.00 | 0.00 | 0.00 | 0.02 | 0.10 | 0.03 | 0.00 | 0.00 | 0.00 |
| Catonella | 0.00 | 0.00 | 0.00 | 0.00 | 0.00 | 0.00 | 0.00 | 0.00 | 0.00 | 0.00 | 0.02 | 0.00 | 0.00 | 0.00 | 0.00 |
| Lachnospiraceae | 0.00 | 0.08 | 0.12 | 0.11 | 0.19 | 0.00 | 0.00 | 0.11 | 0.00 | 0.00 | 0.00 | 0.00 | 0.00 | 0.00 | 0.00 |
| Oribacterium | 0.09 | 0.13 | 0.25 | 0.64 | 0.39 | 0.20 | 1.36 | 0.27 | 0.52 | 0.13 | 0.09 | 0.60 | 0.00 | 0.03 | 0.01 |
| Stomatobaculum | 0.00 | 0.00 | 0.00 | 0.00 | 0.00 | 0.00 | 0.28 | 0.02 | 0.10 | 0.00 | 0.00 | 0.00 | 0.10 | 0.00 | 0.13 |
| Filifactor | 0.00 | 0.00 | 0.00 | 0.00 | 0.00 | 0.00 | 0.00 | 0.00 | 0.00 | 0.01 | 0.16 | 0.09 | 0.00 | 0.00 | 0.00 |
| Peptostreptococcus | 0.00 | 0.00 | 0.00 | 0.00 | 0.28 | 0.00 | 0.17 | 0.24 | 0.08 | 0.00 | 0.36 | 0.10 | 0.00 | 0.12 | 0.00 |
| Bulleidia | 0.00 | 0.00 | 0.00 | 0.00 | 0.00 | 0.10 | 0.00 | 0.00 | 0.00 | 0.00 | 0.00 | 0.00 | 0.00 | 0.00 | 0.00 |
| Solobacterium | 0.00 | 0.00 | 0.04 | 0.15 | 0.24 | 0.10 | 0.20 | 0.22 | 0.73 | 0.00 | 0.13 | 0.18 | 0.19 | 0.47 | 0.45 |
| Anaeroglobus | 0.00 | 0.00 | 0.00 | 0.00 | 0.00 | 0.14 | 0.00 | 0.00 | 0.00 | 0.00 | 0.00 | 0.00 | 0.00 | 0.00 | 0.00 |
| Dialister | 0.00 | 0.00 | 0.00 | 0.00 | 0.00 | 0.02 | 0.00 | 0.00 | 0.00 | 0.00 | 0.00 | 0.00 | 0.00 | 0.00 | 0.00 |
| Megasphaera | 0.00 | 0.00 | 0.00 | 0.22 | 0.09 | 0.00 | 1.15 | 0.04 | 2.88 | 0.00 | 0.19 | 0.06 | 1.30 | 0.28 | 1.61 |
| Mitsuokella | 0.00 | 0.00 | 0.00 | 0.00 | 0.00 | 0.00 | 0.00 | 0.18 | 0.00 | 0.00 | 0.00 | 0.00 | 0.00 | 0.00 | 0.00 |
| Selenomonas | 0.00 | 0.00 | 0.00 | 0.00 | 0.00 | 0.00 | 0.02 | 0.00 | 0.00 | 0.05 | 0.10 | 0.60 | 0.00 | 0.00 | 0.00 |
| Veillonella | 4.55 | 2.93 | 3.13 | 17.87 | 10.95 | 3.78 | 15.51 | 7.17 | 18.98 | 3.08 | 10.42 | 8.92 | 12.48 | 5.62 | 10.47 |
| Fusobacterium | 2.28 | 1.24 | 1.76 | 0.35 | 1.82 | 0.79 | 0.31 | 0.81 | 0.42 | 0.28 | 0.58 | 1.73 | 0.22 | 0.72 | 0.35 |
| Leptotrichia | 0.16 | 0.00 | 0.00 | 0.32 | 0.29 | 0.00 | 0.49 | 0.09 | 0.29 | 0.18 | 0.14 | 0.58 | 1.30 | 0.18 | 0.00 |
| Leptotrichiaceae | 0.54 | 0.17 | 0.49 | 0.46 | 0.66 | 0.08 | 0.79 | 0.13 | 0.18 | 0.11 | 0.00 | 0.34 | 0.86 | 0.79 | 0.56 |
| Lautropia | 0.44 | 1.32 | 2.55 | 0.57 | 1.52 | 2.88 | 2.21 | 4.02 | 3.46 | 6.53 | 3.86 | 1.00 | 0.41 | 2.68 | 0.58 |
| Eikenella | 0.21 | 0.37 | 0.42 | 0.00 | 0.12 | 0.00 | 0.00 | 0.00 | 0.00 | 0.78 | 0.70 | 0.08 | 0.00 | 0.00 | 0.00 |
| Kingella | 0.00 | 0.16 | 0.00 | 0.00 | 0.00 | 0.00 | 0.83 | 0.00 | 0.13 | 0.72 | 0.00 | 0.00 | 0.00 | 0.00 | 0.00 |
| Neisseria | 24.68 | 42.38 | 29.43 | 10.34 | 22.62 | 34.04 | 11.12 | 33.52 | 8.74 | 24.25 | 17.16 | 19.07 | 19.57 | 31.99 | 18.21 |
| Simonsiella | 0.00 | 0.00 | 0.00 | 0.00 | 0.00 | 0.00 | 0.00 | 0.52 | 0.00 | 0.00 | 0.00 | 0.00 | 0.00 | 0.00 | 0.00 |
| Campylobacter | 0.17 | 0.00 | 0.00 | 1.34 | 0.60 | 0.60 | 0.56 | 0.09 | 0.53 | 0.07 | 0.07 | 0.20 | 0.21 | 0.00 | 0.34 |
| Pseudoalteromonadaceae | 0.00 | 0.00 | 0.00 | 0.32 | 0.00 | 0.00 | 0.00 | 0.00 | 0.00 | 0.00 | 0.00 | 0.00 | 0.00 | 0.00 | 0.00 |
| Cardiobacteriaceae | 0.06 | 0.06 | 0.13 | 0.04 | 0.00 | 0.06 | 0.13 | 0.00 | 0.07 | 0.08 | 0.36 | 0.32 | 0.05 | 0.04 | 0.08 |
| Cardiobacterium | 0.31 | 0.19 | 0.48 | 0.03 | 0.00 | 0.00 | 0.00 | 0.00 | 0.00 | 1.14 | 0.53 | 1.12 | 0.00 | 0.06 | 0.00 |
| Escherichia | 0.00 | 0.00 | 0.00 | 0.00 | 0.00 | 0.00 | 0.00 | 0.00 | 0.00 | 0.00 | 0.00 | 0.00 | 2.30 | 0.13 | 0.13 |
| Actinobacillus | 0.00 | 0.00 | 0.00 | 0.00 | 0.00 | 0.00 | 0.11 | 0.10 | 0.00 | 0.14 | 0.00 | 0.00 | 0.00 | 0.26 | 0.00 |
| Aggregatibacter | 0.31 | 0.04 | 1.04 | 0.00 | 0.00 | 0.00 | 1.98 | 0.58 | 0.45 | 0.66 | 1.30 | 0.94 | 0.00 | 0.03 | 0.02 |
| Haemophilus | 10.96 | 9.12 | 13.26 | 5.20 | 9.41 | 7.25 | 5.16 | 9.04 | 5.06 | 11.38 | 6.25 | 5.00 | 4.73 | 13.81 | 8.69 |

**Table 2.**

Follow-up study (P). Relative abundance of TEM-1 genes. XA-Extraction with Antibiotics, b. X-Extraction without Antibiotics. c. C-Control group. B-Baseline, 1W-One week, 2W-Two weeks, 3W-Three weeks, 1M-One month, 3M-Three months, 6M-Six months.

for the control group at Six months.

| **Report** | | | | | | | | |
| --- | --- | --- | --- | --- | --- | --- | --- | --- |
| Group | | B | @1M | @3M | @6M | @1M_B | @3M_B | @6M_B |
| C | N | 10 | 10 | 10 |  | 10 | 10 |  |
|  | Mean | 3.7827 | .8538 | .9992 |  | -2.9290 | -2.7835 |  |
|  | Std. Deviation | 5.21127 | 1.19080 | 1.05553 |  | 5.34650 | 4.37457 |  |
|  | Median | 1.3868 | .3509 | .7898 |  | -.0698 | -.5098 |  |
|  | Minimum | .02 | .06 | .10 |  | -14.14 | -10.83 |  |
|  | Maximum | 14.36 | 3.97 | 3.53 |  | 1.53 | .84 |  |
| E | N | 16 | 16 | 16 | 16 | 16 | 16 | 16 |
|  | Mean | 1.3358 | .6923 | .5348 | 3.2653 | -.6435 | -.8009 | 1.9296 |
|  | Std. Deviation | 3.24407 | 1.08745 | .66261 | 7.88646 | 2.47138 | 3.25324 | 8.89554 |
|  | Median | .0976 | .1991 | .1929 | .1324 | .0363 | .1086 | -.0171 |
|  | Minimum | .01 | .03 | .06 | .00 | -7.69 | -9.67 | -9.36 |
|  | Maximum | 10.70 | 3.01 | 2.43 | 28.80 | 2.67 | 2.36 | 28.70 |
| EA | N | 15 | 15 | 15 | 15 | 15 | 15 | 15 |
|  | Mean | .6699 | .2453 | .7146 | 4.8382 | -.4246 | .0446 | 4.1683 |
|  | Std. Deviation | 1.15995 | .27101 | 1.37094 | 8.49649 | 1.09452 | 1.81575 | 8.75768 |
|  | Median | .1718 | .1297 | .1814 | .1030 | -.0278 | .0096 | .0368 |
|  | Minimum | .01 | .04 | .02 | .00 | -3.65 | -3.29 | -4.20 |
|  | Maximum | 4.28 | .92 | 5.49 | 26.74 | .52 | 5.44 | 26.62 |
| Total | N | 41 | 41 | 41 | 31 | 41 | 41 | 31 |
|  | Mean | 1.6890 | .5681 | .7139 | 4.0264 | -1.1208 | -.9751 | 3.0128 |
|  | Std. Deviation | 3.47319 | .92405 | 1.05182 | 8.08860 | 3.19868 | 3.26317 | 8.75504 |
|  | Median | .1443 | .1823 | .2252 | .1030 | .0072 | .0363 | -.0061 |
|  | Minimum | .01 | .03 | .02 | .00 | -14.14 | -10.83 | -9.36 |
|  | Maximum | 14.36 | 3.97 | 5.49 | 28.80 | 2.67 | 5.44 | 28.70 |
